# Primary adenocarcinoma in the ileostomy of a woman with familial adenomatous polyposis: a case report and literature review

**DOI:** 10.1186/1752-1947-5-556

**Published:** 2011-11-30

**Authors:** Ahmed Hammad, Raed Tayyem, Peter J Milewski, Shanmugavelu Gunasekaran

**Affiliations:** 1 Withybush General Hospital, Fishguard Road, Haverfordwest, SA61 2PZ, UK; 2 Whittington Hospital, Magdala Avenue, London, N19 5NF, UK

## Abstract

**Introduction:**

Ileal adenomas associated with familial adenomatous polyposis are a common finding. Many recent studies following panproctocolectomy for familial adenomatous polyposis have confirmed the presence of multiple ileal adenomas and an increase in ileal mucosal proliferation. In this study, we present a case of invasive adenocarcinoma arising in a severely dysplastic tubulovillous adenoma in the ileostomy of a patient with familial adenomatous polyposis; also, we present a literature review. To the best of our knowledge, only very few cases have been reported in the literature.

**Case presentation:**

A 59-year-old Caucasian woman developed a primary adenocarcinoma in her ileostomy, complicating the stoma 31 years after its formation.

**Conclusions:**

Primary adenocarcinoma following panproctocolectomy for familial adenomatous polyposis is a very rare clinical entity. The risk of developing adenocarcinoma in those patients increases with time. Patient education and medical examination of the stoma are of paramount importance and should be implemented early with the need of designing a surveillance protocol for early detection and management of ileal adenomas, especially in longstanding stomas.

## Introduction

Ileal adenomas associated with familial adenomatous polyposis (FAP) are a common finding. Many recent studies following panproctocolectomy for FAP have confirmed the presence of multiple ileal adenomas and an increase in ileal mucosal proliferation. The management protocol for FAP is prophylactic colectomy with either restorative proctocolectomy with formation of ileal pouch reservoir or ileorectostomy. Panproctocolectomy and terminal ileostomy were the first-line management option in 1950s. Currently, this procedure is performed only for cases with recurrent rectal or ileal pouch adenocarcinoma. In this study, we present a case of adenocarcinoma in the ileostomy of a patient with FAP; in this case, invasive adenocarcinoma arising in a severely dysplastic tubulovillous adenoma was found. Also, we present a literature review. To the best of our knowledge, only 11 cases in English and one in German [1] have been reported so far.

## Case presentation

A 59-year-old Caucasian woman with a previously diagnosed FAP had total colectomy with ileorectal anastomosis 34 years ago followed three years later by resection of the rectal stump and fashioning of an end ileostomy in her left iliac fossa; neither the operative details nor the pathology reports were available. Her medical history shows that she has multiple medical comorbidities, including severe sero-negative rheumatoid arthritis, lumbar disc herniation, total abdominal hysterectomy and bilateral salpingo-oophorectomy., osteoarthritis in both hips, eczema, chronic obstructive pulmonary disease, renal calculi, and chronic renal disease stage 3.

**Figure 1:**
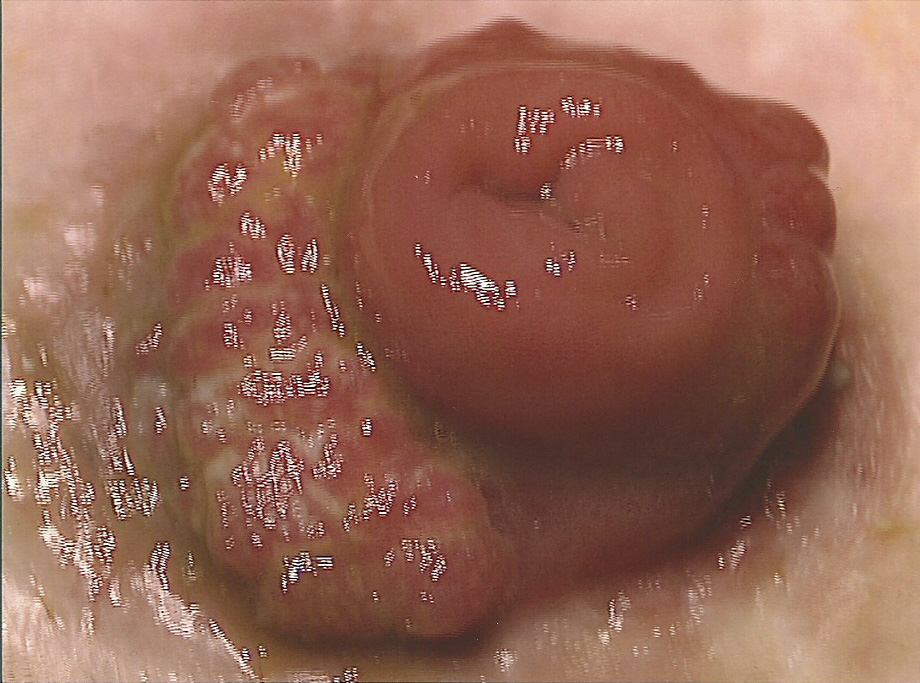
**A fungating growth visible at a stoma**. This fungating growth at an end ileostomy was created 31 years ago, after a panproctocolectomy for familial adenomatous polyposis (FAP). An *en bloc *resection of the stoma showed an invasive and moderately differentiated adenocarcinoma arising in a severely dysplastic tubulovillous adenoma with foci of high-grade dysplasia at the mucocutaneous border.

Three years ago, she was referred to the colorectal clinic because of abnormal fungating growth at the stoma (Figure 1). She was listed for biopsy and diagnostic esophagogastroduodenoscopy (EGD). Pathology from the ileostomy site revealed benign villous adenoma with high-grade epithelial dysplastic changes. The possibility of an invasive focus elsewhere in the lesion could not be excluded. The EGD revealed duodenal polyps, and a biopsy showed tubular adenoma.

A computed tomography scan of her chest, abdomen, and pelvis after oral and intravenous contrast showed no evidence of metastatic disease. Carcinoembryonic antigen was within normal range. After a discussion in the colorectal multi-disciplinary team meeting, a decision was made to keep the duodenal polyps under surveillance and to carry out a barium meal and follow through study. The results of the contrast study were clear; therefore, our patient underwent a laparotomy with *en bloc *small bowel resection with the ileostomy and safety margin of the skin around the stoma. About 45 cm of adherent small bowel was removed alone with the ileostomy specimen. The rest of the small bowel was examined and did not show any gross pathology. A new stoma was created in her right iliac fossa. She went home seven days after a smooth recovery from the operation.

**Figure 2:**
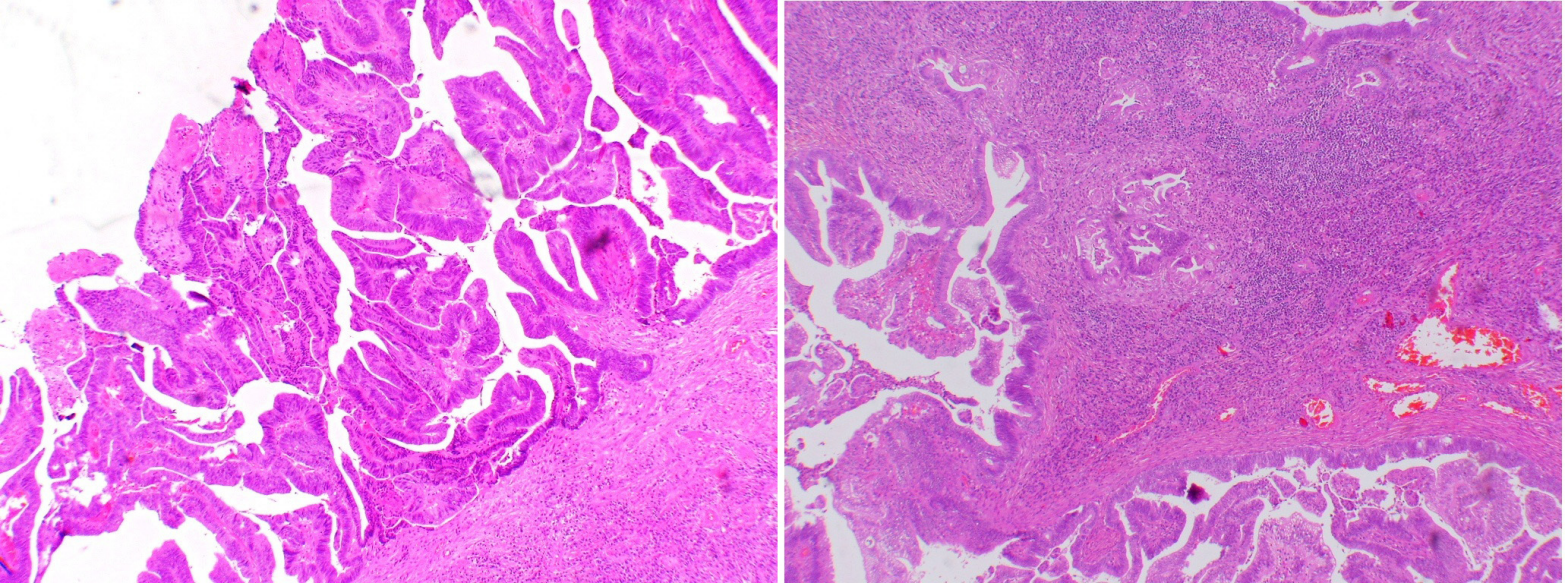
**A picture of the pathology of a polypoid mass at an ileostomy**. Sections from the ileostomy show an invasive and moderately differentiated adenocarcinoma arising in a severely dysplastic tubulovillous adenoma with foci of high-grade dysplasia at the mucocutaneous border.

Pathology showed an invasive, moderately differentiated adenocarcinoma arising in a severely dysplastic tubulovillous adenoma with foci of high-grade dysplasia at the mucocutaneous border (Figure 2). The tumor infiltrated the submucosa and was focally present in the ileostomy stump. The remainder of the resected bowel was clear.

**Figure 3:**
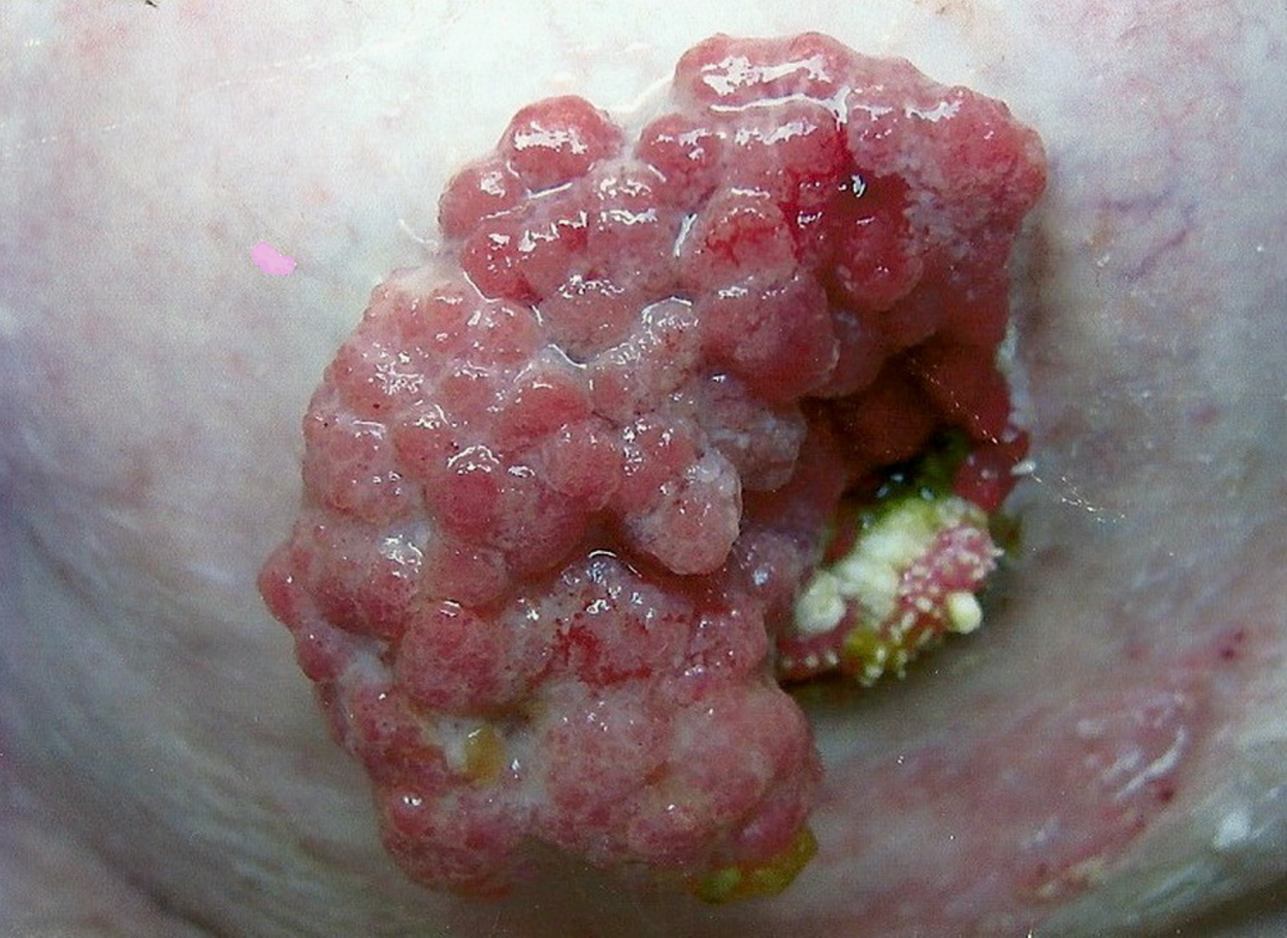
**A recurrent polypoid mass visible at a stoma**. The recurrent polypoid mass is shown at an ileostomy site two years after an *en bloc *resection. A wide excision of the terminal ileum and refashioning of the stoma were undertaken. The specimen was reported as benign tubular adenoma.

As discovered at a follow-up one year ago, she had developed a polypoid mass at the ileostomy site (Figure 3). The results of a limited endoscopic examination of the small bowel up to 50 cm through the stoma were clear. She underwent further wide excision of the terminal ileum and refashioning of the stoma. The specimen was reported as benign tubular adenoma. The follow-up plan was to review our patient every six months in the clinic with an EGD examination every six months.

## Discussion

FAP is an autosomal dominant inherited disease characterized by the presence of hundreds of adenomatous polyps in the colon and rectum. The annual reported incidence ranges from one in 5000 to one in 17,000 live births [2]. The incidence of small bowel malignancy is very low in the general population (seven per 1,000,000) [3]. Suarez and colleagues [4] estimated the incidence of the adenocarcinoma in all ileostomies in Britain to be two to four per 1000, indicating that ileostomies are more prone to malignant changes. In addition, many cases of adenoma in the ileal pouch after proctocolectomy for FAP have been reported and some of them have progressed to adenocarcinoma [5-7].

Many factors have been implicated in the malignant transformation of the ileostomy mucosa: colonic metaplasia and dysplasia of ileal mucosa, chronic irritation due to either physical or chemical trauma, ileitis, and development of chronic inflammatory polyps and malignant transformation in a pre-existing benign adenoma. In this case, we believe that the invasive adenocarcinoma was secondary to malignant transformation in a pre-existing adenoma as evident by the pathology report.

Table 1 summarizes the case reports that we were able to find. There were 13 patients (seven men and six women), and the median age was 57 years (range of 42 to 75). The median interval between stoma formation and presenting adenocarcinoma was 25 years (range of nine to 42). None of the patients had lymph node metastasis at the time of presentation. Three patients showed local skin invasion (23%). No deaths were recorded after three years of follow-up. However, two patients had a recurrence that was excised locally. The treatment was constant in all cases and included *en bloc *resection of the terminal ileum with its mesentery, surrounding stoma skin, and relocation of a new ileostomy. Our patient was followed up to 18 months without evidence of malignant recurrence. However, she developed a benign adenomatous polyp, which was locally excised with refashioning of the stoma.

**Table 1:** Primary adenocarcinoma of an ileostomy in familial adenomatous polyps

Study	Age, years	Sex	Duration, years	Tumor description	Lymph nodes	Pathology	Follow-up
Roth and Logio [8] (1982)	44	Male	9	Ulcerating polypoid mass 2 × 3 cm	N	Moderately differentiated adenocarcinoma involving the ileostomy skin	Not stated
Ross *et al*. [9] (1987)	56	Female	32	Raised mass 12 × 12 cm	N	Mucinous adenocarcinoma	22 months
Primrose *et al*. [10] (1988)	72	Female	25	Friable mass	N	Well-differentiated adenocarcinoma	two years
Suarez *et al*. [4] (1988)	40	Male	29	Polypoid mass	N	Moderately differentiated adenocarcinoma with mucocutaneous invasion	one year
Gilson and Sollenberger [11] (1992)	69	Male	40	Mass 2 × 5 cm	N	Moderately differentiated mucinous adenocarcinoma	two years
Johnson *et al*. [12] (1993)	65	Male	25	Polypoid exophytic mass 3.5 × 3 cm	N	Well-differentiated adenocarcinoma	one year
Lux *et al*. [1] (1993)	42	Male	15	Mass 12 × 12 cm	N	Well-differentiated adenocarcinoma	Not stated
Mimura *et al*. [13] (1999)	54	Male	21	Polypoid mass 6 × 4 × 2 cm	N	Well-differentiated adenocarcinoma	three years
Iizuka *et al*. [14] (2002)	55	Female	14	Polypoid mass extending from 1 to 9 o'clock	N	Well-differentiated adenocarcinoma	23 months
Hata *et al*. [15] (2003)	57	Female	42	Circumferential cauliflower-like polyps	N	Well-differentiated to moderately differentiated adenocarcinoma in adenoma	two years
Shenoy and Cassim [16] (2009)	60	Male	24	Polypoid mass	N	Adenocarcinoma in tubulovillous adenoma	Not stated
Matsushima [17] (2009)	75	Female	Not stated	Fungating mass	N	Well-differentiated adenocarcinoma	32 months
Present case (2010)	59	Female	34	Circumferential polypoid neoplasm 4.5 cm	N	Moderately differentiated mucinous adenocarcinoma infiltrating the deep dermis	one year

This table summarizes 13 case reports of primary adenocarcinoma of an ileostomy in familial adenomatous polyps. N, negative.

## Conclusions

The number of reported cases for primary adenocarcinoma following panproctocolectomy for FAP is small. Nevertheless, it is expected that the numbers will increase in longstanding ileostomies in parallel with the duration of the stoma. Patient education and medical examination of the stoma should be implemented early with the need of designing a surveillance protocol for early detection and management of ileal adenomas, especially in longstanding stomas. Surgery should include an *en bloc *resection of the ileum with a generous margin of the surrounding skin and relocation of the stoma.

## Abbreviations

EGD: esophagogastroduodenoscopy; FAP: familial adenomatous polyposis.

## Consent

Written informed consent was obtained from the patient for publication of this case report and any accompanying images. A copy of the written consent is available for review by the Editor-in-Chief of this journal.

## Competing interests

The authors declare that they have no competing interests.

## Authors' contributions

AH performed literature searches, wrote the paper, and assisted in the operation. RT contributed in writing the amendments and in revising the manuscript. PJM and SG performed the operation and supervised the final revision of the paper. All authors read and approved the final manuscript.

## References

[B1] Lux N Wedell J Busch M van Calker H Adenocarcinoma of the ileostomy after total proctocolectomy in familial polyposis. A case report and synthesis of previously published cases [in German] Chirurg 1993 64 416 418 8392459

[B2] Utsunomiya J Lynch HT Hereditary Colorectal Cancer: International Symposium Proceedings 1990 Berlin: Springer-Verlag Berlin and Heidelberg GmbH & Co. K

[B3] Barclay TH Schapira DV Malignant tumors of the small intestine Cancer 1983 51 878 881 10.1002/1097-0142(19830301)51:5<878::AID-CNCR2820510521>3.0.CO;2-V 6821853

[B4] Suarez V Alexander-Williams J O'Connor HJ Campos A Fuggle WJ Thompson H Enker WE Greenstein AJ Carcinoma developing in ileostomies after 25 or more years Gastroenterology 1988 95 205 208 3371615 10.1016/0016-5085(88)90313-7

[B5] Tajika M Nakamura T Nakahara O Kawai H Komori K Hirai T Kato T Bhatia V Baba H Yamao K Prevalence of adenomas and carcinomas in the ileal pouch after proctocolectomy in patients with familial adenomatous polyposis J Gastrointest Surg 2009 13 1266 1273 10.1007/s11605-009-0871-1 19333660

[B6] Parc YR Olschwang S Desaint B Schmitt G Parc RG Tiret E Familial adenomatous polyposis: prevalence of adenomas in the ileal pouch after restorative proctocolectomy Ann Surg 2001 233 360 364 10.1097/00000658-200103000-00009 11224623 PMC1421251

[B7] Friederich P de Jong AE Mathus-Vliegen LM Dekker E Krieken HH Dees J Nagengast FM Vasen HF Risk of developing adenomas and carcinomas in the ileal pouch in patients with familial adenomatous polyposis Clin Gastroenterol Hepatol 2008 6 1237 1242 10.1016/j.cgh.2008.06.011 18848811

[B8] Roth JA Logio T Carcinoma arising in an ileostomy stoma: an unusual complication of adenomatous polyposis coli Cancer 1982 49 2180 2184 10.1002/1097-0142(19820515)49:10<2180::AID-CNCR2820491033>3.0.CO;2-3 6210427

[B9] Ross DS Bussing R Dietrich J Carcinoma arising in an ileostomy IMJ Ill Med J 1987 172 163 166 2889705

[B10] Primrose JN Quirke P Johnston D Carcinoma of the ileostomy in a patient with familial adenomatous polyposis Br J Surg 1988 75 384 10.1002/bjs.1800750430 2833981

[B11] Gilson TP Sollenberger LL Adenocarcinoma of an ileostomy in a patient with familial adenomatous polyposis. Report of a case Dis Colon Rectum 1992 35 261 265 10.1007/BF02051020 1310928

[B12] Johnson JA III Talton DS Poole GV Adenocarcinoma of a Brooke ileostomy for adenomatous polyposis coli Am J Gastroenterol 1993 88 1122 1124 8391212

[B13] Mimura T Kuramoto S Yamasaki K Kaminishi M Familial adenomatous polyposis: a case report and histologic mucin study J Clin Gastroenterol 1999 28 372 376 10.1097/00004836-199906000-00021 10372942

[B14] Iizuka T Sawada T Hayakawa K Hashimoto M Udagawa H Watanabe G Successful local excision of ileostomy adenocarcinoma after colectomy for familial adenomatous polyposis: report of a case Surg Today 2002 32 638 641 10.1007/s005950200116 12111524

[B15] Hata K Watanabe T Kawamura YJ Ishigami H Kanazawa T Tada T Zhao B Koketsu S Nagawa H K-ras mutation and loss of heterozygosity at 17p with beta-catenin accumulation in intramucosal carcinoma of the ileostomy in familial adenomatous polyposis: a case report Dig Dis Sci 2003 48 2310 2314 14714618 10.1023/b:ddas.0000007868.52339.22

[B16] Shenoy S Cassim R Ileostomy adenocarcinoma associated with familial adenomatous polyposis (FAP): new problem in old disease Int J Colorectal Dis 2009 24 1475 1476 10.1007/s00384-009-0739-6 19488768

[B17] Matsushima K Two different types of polypoid lesion at ileostomy site Surgery 2009 145 337 338 10.1016/j.surg.2007.08.021 19231588

